# Virulence of a T6SS *Campylobacter jejuni* chicken isolate from North Romania

**DOI:** 10.1186/s13104-019-4201-8

**Published:** 2019-03-28

**Authors:** Vlad A. Ungureanu, Alexandros Ch. Stratakos, Ozan Gundogdu, Lavinia Stef, Ioan Pet, Elena Pet, Nicolae Pacala, Nicolae Corcionivoschi

**Affiliations:** 1 School of Bioengineering and Animal Resources, Banat University of Animal Sciences and Veterinary Medicine, King Michael I of Romania, Timisoara, Romania; 2 Bacteriology Branch, Veterinary Sciences Division, Agri-Food and Biosciences Institute, Newforge Lane, Belfast, BT9 5GB Northern Ireland UK; 3 Auranta, NovaUCD, Dublin, Ireland; 4 Faculty of Infectious & Tropical Diseases, London School of Hygiene and Tropical Medicine, 11 Keppel Street, London, WC1E 7HT UK; 5 School Management and Rural Tourism, Banat University of Animal Sciences and Veterinary Medicine, King Michael I of Romania, Timisoara, Romania

**Keywords:** *Campylobacter*, Virulence, T6SS, Chicken isolates

## Abstract

**Objectives:**

In this study we have investigated the in vitro and in vivo virulence characteristics of a new T6SS positive *Campylobacter jejuni* chicken isolate (SV12) originating from a poultry population in North Romania. A detailed phenotypic characterization was performed and compared to the T6SS negative *C. jejuni* 81–176 wild strain.

**Results:**

Our results indicate that the significantly higher capacity to attach and invade HCT-8 cells of *C. jejuni* SV12 isolate is associated with increased motility, increased resistance to bile salts and serum resistance, when compared to *C. jejuni* strain 81–76. Mice infected with the SV12 isolate showed statistically higher levels of colonization at both 7- and 14-days post-inoculation and in the stomach, caecum, duodenum and large intestine. Infection with the SV12 strain induced a stronger immune response as the gene transcript levels of IL-17, TNFα and IFNγ were more pronouncedly up-regulated compared to the *C. jejuni* strain 81–176. The present study showed that the new isolate SV12 had an enhanced virulence capacity compared to the wild strain which was evident in vivo as well. This work also provides an insight on the colonization pattern and host immune response differences between T6SS positive and T6SS negative *C. jejuni*.

## Introduction

Campylobacters are responsible for a wide range of illnesses including diarrhoea, reactive arthritis and leading in some cases to serious neuromuscular syndromes such as Guillain-Barré [1]. In some instances, *Campylobacter* spp., can produce and secrete toxins which can be transferred into eukaryotic [2] or prokaryotic cells. The transfer of these toxins can take place via the novel Type VI secretion system (T6SS), reported in Campylobacter recently [3, 4]. The identity component of the T6SS is the presence of the hcp gene [5]. The presence of this gene has been used to associate the T6SS system with severe infections in humans having as the most common symptoms bloody diarrhoea [6]. It has also been shown that the hcp^+^ positive campylobacters display better adhesion and invasion properties in vivo [7, 8].

*Campylobacter jejuni* readily colonizes poultry within the first 12–14 days of life and is considered a commensal with no disease phenotype and as consequence will not provide an insight into *C. jejuni* [9]. Mice would normally provide a preferred infection model system for pathogenic infection by *C. jejuni* especially if the aim is to investigate its colonization abilities only [10]. The aim of this study was to investigate the in vitro and in vivo virulence capacity of a T6SS *Campylobacter* isolate (SV12). This isolate originates from the poultry populations in North Romania (Moldova).

## Main text

### Materials and methods

#### Bacterial strains and growth conditions and T6SS detection

*Campylobacter jejuni* strain (SV12) was isolated from caecal content broilers, conventionally housed raised in small family farms within the regions of Moldova, Romania. The isolate SV12 was retained and detected positive for T6SS [11]. *C. jejuni* NCTC 12502 was used as the positive control. *C. jejuni* 81–176 was used as control strain in the infection assays.

#### In vivo infection assay

The infection assay was performed as previously described [12] using 5- to 6-week-old BALB/c mice (n = 10) with normal gut flora were purchased from Charles River Laboratories. All experiments were approved by the Animal Research Committee according to the legislation in place (Law 471/2002 and government ordinance 437/2002) and under the supervision of National Sanitary Veterinary Agency. The ethics committee of Banat University of Agricultural Sciences and Veterinary Medicine—King Michael I of Romania, approved this work.

#### Infection assays

The gentamicin protection assay was used to test the ability of *C. jejuni* SV12 chicken isolates to adhere and invade human intestinal epithelial cells using as control *C. jejuni* 81–176 as previously described [6]. The experiments were conducted on three separate occasions. The significance of differences in adhesion and invasion between samples was determined using the Student t test. A p value of < 0.05 was defined as significant.

#### Motility assays and serum resistance

In order to investigate the motility and serum resistance of the SV12 isolate we have used methodologies previously published [6, 13]. The sensitivity of bacteria to human serum (Invitrogen) was measured by adding 5 μl of bacterial suspension to duplicate wells of a six-well plate containing 800 μl of Mueller–Hinton broth and 200 μl of active pooled human serum. All assays were conducted in triplicate and repeated independently three times.

#### Resistance to bile salts

The resistance to bile salts was investigated as previously described [14]. These experiments were done in triplicates. Viable cell counts for each plate were determined at the end of the incubation period.

#### Antimicrobial resistance

The levels of antibiotic resistance for *C. jejuni* SV12 was tested by using nalidixic acid, ciprofloxacin, erythromycin, ampicillin, amoxicillin-clavulanic acid and gentamicin as previously described [14]. The test used was the disk E test from Solna, Sweeden.

#### RNA extraction and quantitative real-time PCR

Tissue samples isolated from the infected or control mice were preserved in RNAlater at − 20 °C for later use. RNA was extracted using a Qiagen RNeasy kit (Qiagen) according to the manufacturer’s protocol. The primers used have been described previously described [15].

#### Statistical analysis

Experiments were conducted on at least three biological replicates. Results are presented as the means ± standard deviations (error bars) of three replicate experiments. Graphs were drawn using Prism, and the unpaired Student t test was used to estimate statistical significance. A p value of < 0.05 was considered significant.

### Results

#### In vitro virulence assay to test the virulence of the SV12 isolate

**Fig. 1:**
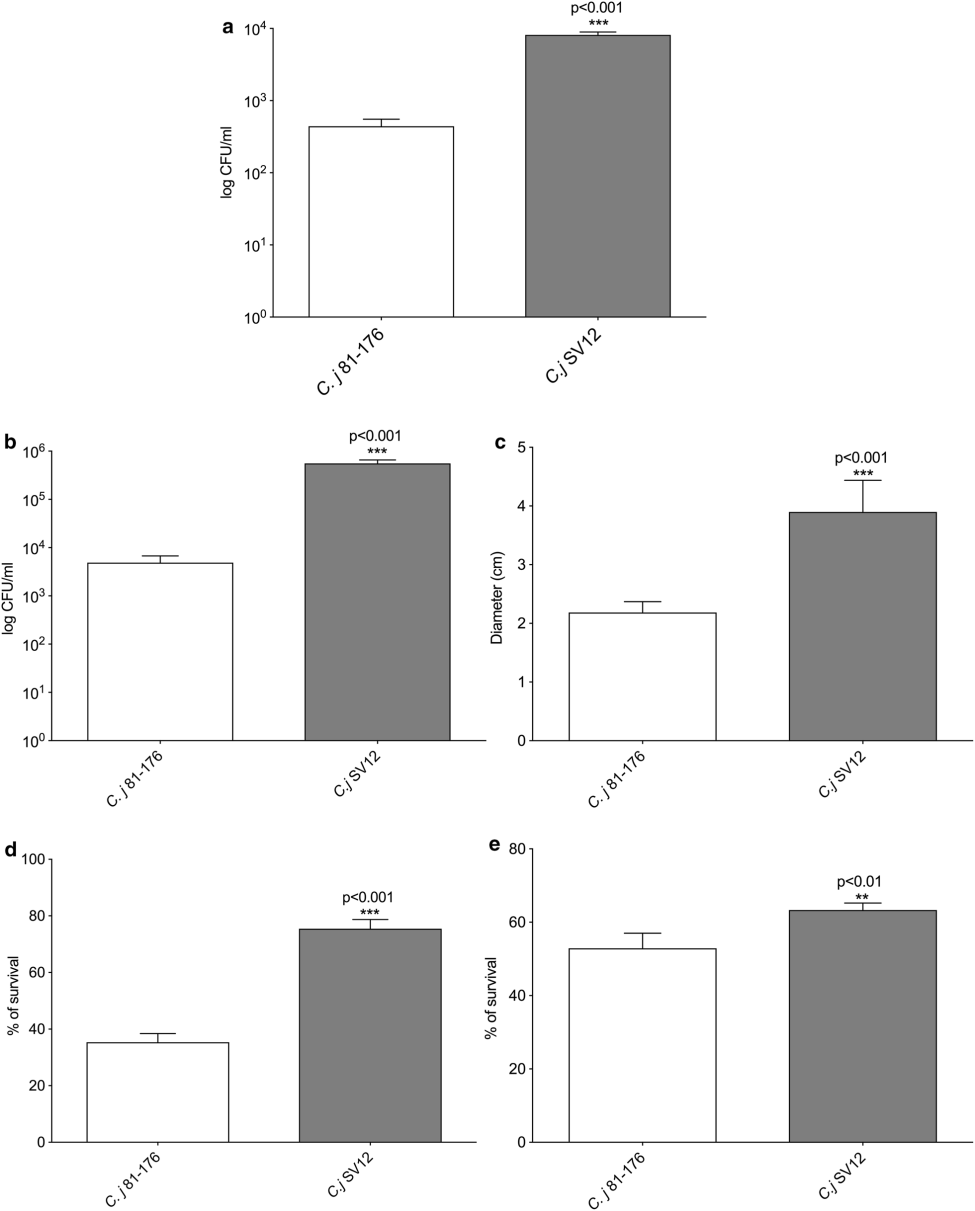
Adhesion and invasion of the SV12 isolate. **a** Adhesion, **b** invasion of HCT-8 cells of *C. jejuni* 81–176 and *C. jejuni* SV12. Statistical significance (Student’s *t* test) relative to the level of *C. jejuni* 81–176 strain is indicated. The experiments were done in triplicate and on three separate occasions. The error bars represent standard deviations for six separate wells. **c** Motility of the *C. jejuni* chicken isolate SV12 and the *C. jejuni* 81–176 strain; **d** serum resistance is characterized as the number of *C. jejuni* colonies enumerated following exposure to human serum and divided by the number of colonies surviving to heat-inactivated serum (%) and **e** resistance to bile salts. Results are the mean of three separate experiments. Statistical significance (Student’s *t* test) relative to the level of *C. jejuni* 81–176 strain is indicated

The T6SS marker of SV12 isolate was detected as described in “Materials and methods” (data not shown) and its virulence aptitudes (invasion, adhesion) were confirmed using HCT-8 cells. Invasiveness varied between the investigated strain, as is shown in Fig. 1a (adhesion) and 1B (invasion). Following gentamicin protection assay we have shown that the total adhesion of the chicken isolate is significantly increased compared to the total adhesion of *C. jejuni* 81–176. The *C. jejuni* chicken SV12 had higher levels of internalization compared to the *C. jejuni* 81–176. Similarly, to the adhesion results, the chicken isolate SV12 had the highest internalization levels compared to *C. jejuni* 81–176.

#### Investigation of SV12 bacterial motility and serum resistance

**Fig. 2:**
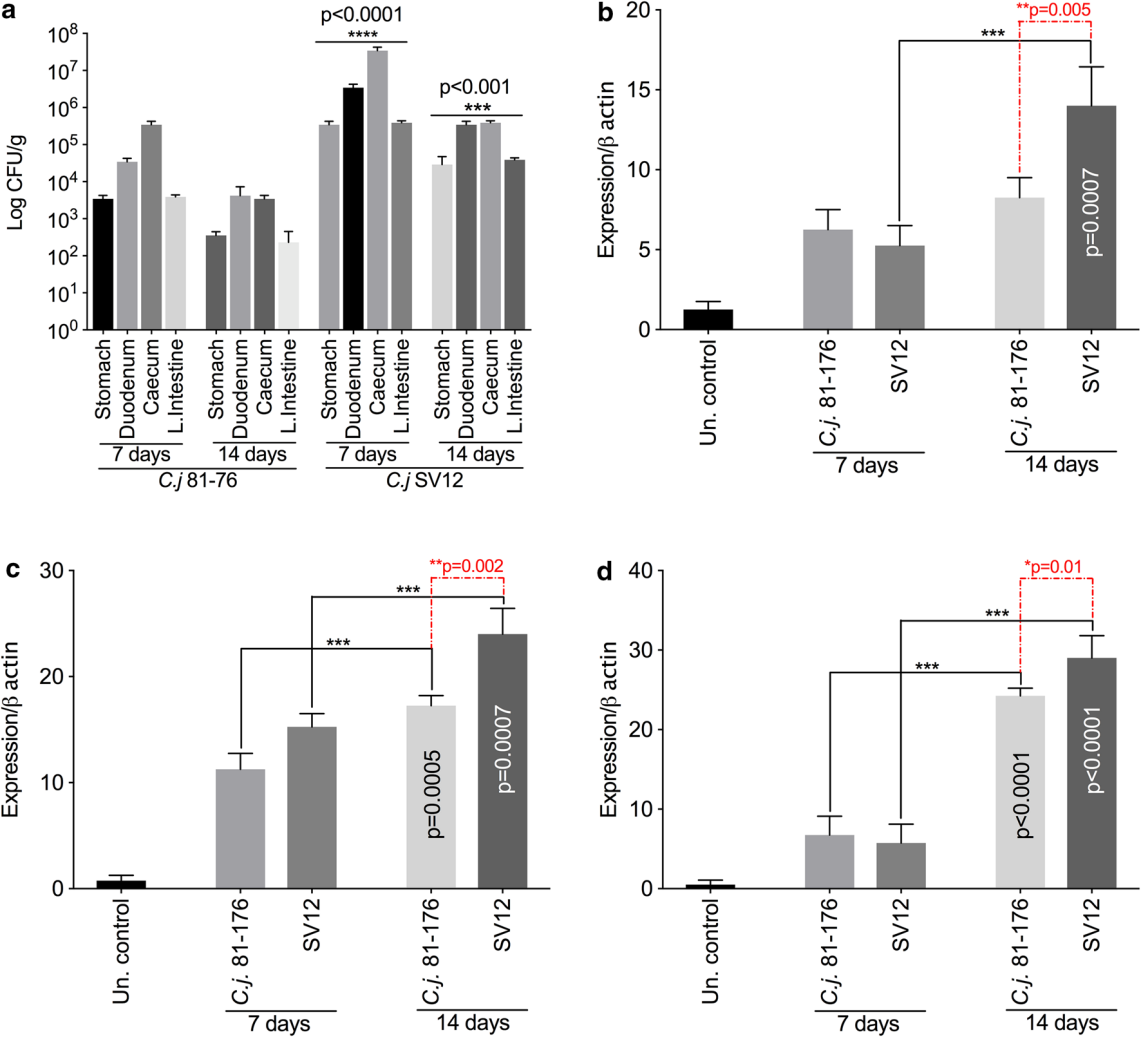
Motility, serum and bile resistance of *C. jejuni* SV12 isolate. For bacterial count determination of *C. jejuni* 81–176 and *C. jejuni* SV12 groups of 12 mice were tested as described in “Materials and methods” **a** in the stomach, duodenum, large intestine and small intestine. RT-qPCR was used to investigate the cytokine production in the RNA extracted from the ceca of uninfected control or infected mice at 7- and 14-days post infection. **b** IL-17, **c** TNFα and **d** IFNγ. Statistical significance was determined using a student *t* test

We have next investigated the role of motility, as virulence factor, in the pathogenicity of the SV12 isolate. Our results show that its motility was significantly increased (4 cm, p < 0.001) when compared to the *C. jejuni* 81–176 wild-type strain (2 cm) as observed in semisolid agar (Fig. 1c). The investigation of serum resistance abilities revealed that the survival rates of this new chicken isolate, SV12 are improved. As shown in Fig. 2b, the SV12 isolate had a rate of survival of 78% (p < 0.001) compared to a survival rate of only 38% for *C. jejuni* 81–176. These results suggest that the increase in motility could account for the increase in the adhesion/invasion of this isolate (Fig. 1). Moreover, the increased rate of survival in human serum indicates the potential of *C. jejuni* SV12 to cause systemic infections.

#### In vitro resistance of SV12 isolate to bile salts

The ability to cope with the bactericidal activity of the bile is key for *C. jejuni* and to colonize the human or the animal gastrointestinal tract. We aimed to measure the response of *C. jejuni* 81–176 and SV12 isolate to the presence of bile salts in the culture media. The chicken isolates SV12 had significant (p < 0.01) better survival rate (65%) compared to only 55% survival rate of *C. jejuni* 81–176 (Fig. 1d). Similar to the results observed for the survival to human serum and motility the resistance to bile salts also indicates a possible higher virulence aptitude for the SV12 isolate (Fig. 1e).

#### Antimicrobial resistance

For SV12 isolate and the control strain *C. jejuni* 81–176 the antibiotic inhibition zones to the various antibiotics were investigated. The antibiotic profile of the *C. jejuni* SV12 isolate was clearly different to *C. jejuni* 81–176 which showed resistance only to gentamicin (Table 1). The chicken isolate SV12 was sensitive to all antibiotics except to amoxicillin–clavulanic acid.

**Table 1:** Antimicrobial resistance rates of *C. jejuni* isolated from poultry

Isolate	Nalidixic acid	Ciprofloxacin	Erythromycin	Ampicillin	Amoxicillin–clavulanic acid	Gentamicin
*C. jejuni* 81–176	−	−	−	−	−	+
SV12	+	+	+	+	−	+

#### In vivo colonization abilities of *C. jejuni* SV12 and immune response in infected mice

The in vitro experiments suggested a higher virulence ability of the SV12 isolate. In order to get a better understanding, we have performed in vivo mice infections to get a better insight on its pathogenicity. *C. jejuni* is usually detected in the large intestine and caecum in mice (n = 10) for each experimental group including the un-infected control. In our experiments *C. jejuni* 81–176 wild-type strain and the SV12 isolate were well able to colonize the stomach, duodenum, large intestine and small intestine (Fig. 2a). However, in all cases the mice infected with the SV12 isolate displayed higher and statistically significant levels at 7 days (p < 0.0001) and also at 14 days (p < 0.001) post-inoculation and in all compartments of the gut investigated (Fig. 2a). The higher colonization rates were associated with an increase in the immune response to infection. The investigation of gene transcript levels of IL-17 (Fig. 2b), TNFα (Fig. 2c) and IFNγ (Fig. 2d) suggested gut layer inflammation in infected mice as they showed upregulated gene transcript levels for all these three cytokines in mice infected with the SV12 isolate. All these elevated levels of cytokine release were statistically significant as described in Fig. 2.

### Discussion

Motility allows *Campylobacter* to penetrate the mucus so it can adhere to and invade surface epithelial cells [14]. In this study, the motility of the *C. jejuni* isolate SV12 and *C. jejuni* 81–176 wild-type strain was determined and compared. SV12 showed significantly higher motility which was also consistent with a higher attachment capacity exhibited compared to *C. jejuni* 81–176 wild-type strain. Differences in motility are of great importance during infection and can influence the pathogen’s ability to cause disease.

SV12 also showed significantly higher internalization in HCT-8 cells. Cell adhesion and invasion are critical for gut pathogens to exert their pathogenic potential and establishing persistence. Recent studies on *E. coli* suggest that T6SS promotes host cell adhesion and invasion, the two fundamental steps required for host colonization and the attainment of full virulence [16]. It is possible that *C. jejuni hcp* possibly serves as a structural protein that can facilitate host cell adhesion as well as an effector that can promote host cell invasion [7]. Therefore, the higher adhesion and invasion levels observed here could be attributed to the fact that SV12 harbours a functional T6SS system. *C. jejuni* must be able to withstand the bile salts secreted into the intestinal tract and the bactericidal effects of the human and animal serum in order to cause infection [17]. In the present study, although both strains were susceptible to bile salts, SV12 showed increased survival compared to the *C. jejuni* 81–176 control strain. The same trend was also evident when both strains were exposed to human serum.

*Campylobacter* can acquire antimicrobial resistance genes via horizontal gene transfer, acquisition of mobile genetic elements and mutations. Treatment of severe infection occasionally necessitates antimicrobial therapy. Although fluoroquinolones were regularly used in the past to fight the infection, the rising of resistance among *Campylobacter* isolates has rendered these antibiotics ineffective and now erythromycin is the antibiotic of choice [18]. The present study showed that the SV12 was more resistant to nalidixic acid, ciprofloxacin, erythromycin, and ampicillin compared to the wild strain. The study of According to Lluque et al. there is an increasing trend in macrolide resistance quinolones resistance, among *C. jejuni*. Stressing the need of new antimicrobial agents to treat infections cause by this pathogen [19].

The above results show that the *C. jejuni* chicken isolate SV12 exhibits an enhanced range of virulence phenotypes in vitro compared to the wild strain. Nevertheless, extrapolating these results to the host should be done with caution as the host’s susceptibility plays an important role in *C. jejuni* infection [20]. In order to confirm the increased virulence potential of *C. jejuni* SV12 an in vivo infection using a mouse model was also performed. The results revealed that *C. jejuni* multiplied in the intestines of mice. The caecum and the duodenum having the highest levels of *C. jejuni* both after 7- and 14-days post inoculation. Although both *C. jejuni* isolates used in this study were able to colonise the intestine, their ability to colonize the intestines varied. The strain which was more virulent based on the results of the in vitro studies (SV12) showed higher levels in the stomach, duodenum, caecum and large intestine compared to the wild strain.

The cytokine profile was also determined in order to investigate the host immune response to the two *C. jejuni* strains. The results revealed that *C. jejuni* infection resulted in up-regulation of IL17, TNFα and IFNγ mRNA expression. Mice infection with a human and food *C. jejuni* isolates induced strong pro-inflammatory responses with elevated levels of IL-12, TNF-α and IFN-γ. Cytokine IL-17 has also been found to increase in the intestines of *C*. *jejuni* infected mice [21, 22]. The results here show that host immune response to *C. jejuni* differs between *hcp* positive and *hcp* negative strains, with the *hcp* positive strain inducing a stronger immune response.

## Limitations

The study has one major limitation it did not include additional *C. jejuni* strains. The inclusion of more poultry isolates, both T6SS positive and T6SS negative, would allow to further confirm the in vitro and in vivo differences in virulence, observed.

## Abbreviations

T6SS: type six secretion system
IL17: interleukin 17A
TNFα: tumor necrosis factor alfa
IFNγ: interferon gamma
